# Orally Administered CBD/CBG Hemp Extract Reduces Severity of Ulcerative Colitis and Pain in a Murine Model

**DOI:** 10.3390/jcm14176095

**Published:** 2025-08-28

**Authors:** Shivani S. Godbole, Dongxiao Sun, Matthew D. Coates, Victoria J. Himmelberger, Diana E. Roopchand, Wesley M. Raup-Konsavage

**Affiliations:** 1 Department of Neuroscience & Experimental Therapeutics, Penn State College of Medicine, Hershey, PA 17033, USA; 2 Center for Cannabis & Natural Product Pharmaceutics, Penn State College of Medicine, Hershey, PA 17033, USA; 3 Department of Medicine, Penn State College of Medicine, Hershey, PA 17033, USA; 4 Department of Food Science, Rutgers University, New Brunswick, NJ 08901, USA

**Keywords:** ulcerative colitis, *Cannabis sativa*, cannabigerol, cannabidiol, DSS model in mice

## Abstract

**Background:** Ulcerative colitis (UC) is an autoimmune disorder characterized by inflammation of the mucosa that gives rise to a disrupted epithelial morphology. Persistent or recurrent inflammation and the debilitating nature of the associated symptoms make treatment of UC challenging. Cannabinoids derived from *Cannabis sativa* L. have been used for treatment of gastrointestinal disorders due to the wide-ranging therapeutic benefits of these compounds. **Methods***:* We evaluated a commercial hemp extract, high in cannabigerol (CBG) and cannabidiol (CBD), as a novel treatment for UC symptoms using the dextran sodium sulfate (DSS) model in mice. Hemp extract was administered via two different routes of administration, intraperitoneal (i.p) and oral (p.o). **Results:** Specifically, we observed that cannabinoid treatment reduced damage to the colonic epithelium. We also observed that CBG/CBD rich hemp extracts help reduce pain-related responses in these animals. **Conclusions:** Together, the data suggest that cannabinoid administration has the potential to be an effective alternate therapeutic option for UC management.

## 1. Introduction

Ulcerative colitis (UC) is a multifactorial, chronic disease of the gut characterized by inflammation of the colonic mucosa [1,2,3,4,5]. The associated inflammatory process leads to the alteration and/or loss of epithelial crypts and mucus-producing goblet cells [6,7,8,9]. Over time, this results in a variety of significant symptoms, including weight loss, looser and more frequent stools, and abdominal pain [10,11]. These symptoms are common in UC and challenging to manage even during periods of seeming quiescence [12,13]. They are also important because they diminish patient quality of life while increasing the risk of costly (and potentially unnecessary) medical interventions [14,15].

A large number and variety of medical therapies currently exist to treat UC. However, patients are frequently non-responsive, experience adverse side-effects, and/or lose their response to these medications, making management of UC an ongoing challenge [16,17]. Additionally, as indicated above, UC patients can also experience significant symptoms even when in remission. These persistent challenges necessitate the development of alternate therapeutic options.

Cannabinoids derived from *Cannabis sativa* L. have been reported to have anti-inflammatory properties and have been proposed as potential treatments for a variety of immunological disorders [18,19,20]. Patients suffering from UC have reported using cannabinoids, particularly Δ^9^-tetrahydrocannabinol (THC), for symptom management, including pain [14,21,22,23]. THC activates the ubiquitously expressed cannabinoid 1 receptor (CB_1_R) in the brain to induce antinociception [24,25]. The cannabinoid receptors (CB_1_R and CB_2_R) are G_i/o_ coupled receptors, and their activation leads to inhibition of adenyl cyclase (AC). Downstream signaling of these GPCRs enables them to regulate the release of neurotransmitters (GABA, glutamate, and dopamine), thereby producing the desired antinociception [26,27]. While the CB_1_Rs are located mainly in the CNS, the CB_2_Rs are largely localized on peripheral tissues (immune and lymphatic systems), and CB_2_R activation inhibits the expression of proinflammatory cytokines, particularly IL-12 and IL-23 [28,29].

Recent discoveries underscore the potential of cannabinoids for management of UC and/or its symptoms. For example, several studies suggest that the endocannabinoid system (consisting of cannabinoid receptors, endogenous ligands, and synthesizing enzymes) is involved in the pathophysiology of UC [30,31]. Mice developed aggravated UC symptoms upon knockdown of CB1 and CB2 receptors [32]. In humans, lower circulating levels of endocannabinoids (anandamide and 2-acyl glycerol) were observed in UC patients and were correlated with the presence of certain UC symptoms. Of note, administration of cannabis extracts to these patients helped in providing relief from these symptoms, further corroborating the basis for cannabinoid use in UC management [31].

Previously, we have shown that intraperitoneal administration of a CBD/CBG dominant hemp extract improved ulcerative colitis in a mouse model of UC. To expand on those findings, we utilized a self-administered orally route of administration, enhancing the translatability of our prior findings, and assessed the impact of hemp extract on additional aspects of UC, most notably pain [33]. We find that daily oral administration of hemp extract is as protective as intraperitoneal administration at reducing colitis severity and a proxy of abdominal pain perception in a murine model of UC.

## 2. Materials and Methods

### 2.1. Administration of Hemp Extract (HE)

**Figure 1:**
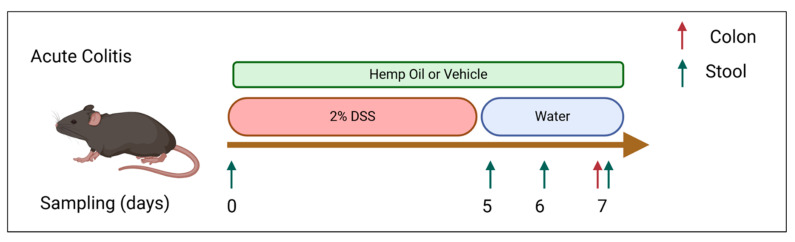
Schematic of experimental design. Colitis is induced using a 2% DSS solution administered via drinking water. Cannabinoids were administered via diet or i.p throughout the course of DSS treatment.

CBD/CBG hemp extract (HE) (Extract Laboratories, Lafayette, CO) was administered at a daily dose of 20 mg/kg CBG and 20.7 mg/kg CBD via intraperitoneal injection as previously described. Fractionated coconut oil (Pursonic, New York, NY, USA) was used for vehicle-treated animals, either i.p. or orally in Nutella. Additionally, a separate group of mice received a daily dose of the same HE at 30 mg/kg CBG, and 31 mg/kg CBD mixed in Nutella for oral administration; this higher dose is to account for differences in pharmacokinetics of cannabinoids between oral and i.p. delivery [34,35,36]. Non-colitic control animals also received a daily dose of Nutella with vehicle (coconut oil) or CBD/CBG HE (Figure 1).

### 2.2. Animals and Induction of Colitis

Six--to-eight-week-old male C57BL/6 (Jackson Laboratories, Bar Harbor, ME, USA) mice were used for this study. Colitis was induced by administration of 2% DSS solution (ThermoFisher, Waltham, MA, USA) in their drinking water for 5 days, followed by returning the animals to normal water for 2 additional days [37,38,39,40]. A second cohort of animals were maintained on normal water during the study to serve as control. Animals were individually housed in a barrier facility and had access to food and water ad libitum. The Pennsylvania State University College of Medicine Institutional Animal Care and Use Committee approved the animal protocols used in this study. According to calculations performed using power analysis, a cohort of 20 mice per experimental group was used to achieve statistical significance.

### 2.3. Mass Spectroscopy

Mice were anesthetized on day 7 of the study, and trunk blood was collected by decapitation. Whole blood was centrifuged at 4 °C at 2000 RPM for 10 min, and plasma was collected and stored at −80 °C until further analysis. Plasma concentrations of CBG and CBD were determined using HPLC-Mass spectroscopy. As previously described, standard curves were constructed by plotting the ratio of the analyte peak area to internal standard peak area vs. analyte concentration [41].

### 2.4. Disease Activity Index (DAI) Scoring

DAI scoring following DSS-treatment was performed, as previously described [33,38,39,40]. Animals were scored daily based on percent weight loss, stool consistency, and presence of blood in stool/rectum, with a score of 0–4 for each category (Table 1).

The three scores are added together for a DAI score, and data are presented as average daily DAI scores.

### 2.5. Tissue Collection and Processing

Animals were euthanized (day 7) via isoflurane overdose followed by decapitation, and trunk blood was collected. Colon tissue was collected and fixed in 3.7% paraformaldehyde overnight and then transferred to 70% ethanol. H&E staining, paraffin embedding, and slide preparation were all performed by the Comparative Medicine Histology Core at Penn State College of Medicine [33]. Damage to the colonic epithelium was measured using ImageJ software (version 1.54p), and the total length of damaged tissue was divided by 2× the total length for that animal for percentage damage estimation.

### 2.6. Von Frey Assay

Mechanical sensitivity was assessed using an electronic Von Frey anesthesiometer (IITC Life Sciences Inc., Woodland Hills, CA, USA). Mice were placed in individual acrylic chambers placed on a wire mesh table. Pain sensitivity was evaluated after a 20 min habituation period to the testing environment. The von Frey anesthesiometer was equipped with a semi-flexi tip (IITC Life Sciences Inc., Woodland Hills, CA, USA), which was applied to the abdominal wall. The perianal and external genitalia areas were avoided while measuring abdominal hypersensitivity. The mechanical stimulation was concentrated on the lower and mid abdomen. Mechanical sensitivity was assessed before beginning DSS treatment to record baseline values, on day 5 when the mice come off DSS treatment, and on day 7, when the inflammation is at its peak. Mechanical sensitivity was recorded for colitic mice that received HE or vehicle administered via i.p or diet [42,43].

### 2.7. Alcian Blue Staining

Alcian blue staining (pH 2.5) was performed to measure the goblet cell density in tissue. Slides were deparaffinized by passing through graded xylene, 100% ethanol, and 95% ethanol and rehydrated by incubating slides in distilled water. Slides were placed in a 3% acetic acid solution for 5 min followed by staining in alcian blue solution (pH 2.5) for 30 min at room temperature and rinsed in water. The tissue was counterstained with nuclear fast red, rinsed and dehydrated using graded alcohols prior to mounting. The slides were examined at 400× magnification. Goblet cell count was measured using ImageJ software.

### 2.8. Statistical Analysis

All data are shown as mean ± standard deviation, with individual data points shown. The number of mice per experiment are indicated in each caption, but a minimum of five mice were used in each experiment. Statistical significance was assessed using Prism software (10.4.1, GraphPad, Boston, MA, USA) using the unpaired, non-parametric *t*-test.

## 3. Results

### 3.1. Oral Administration of CBD/CBG Hemp Extract Reduces Colitis Severity

**Figure 2:**
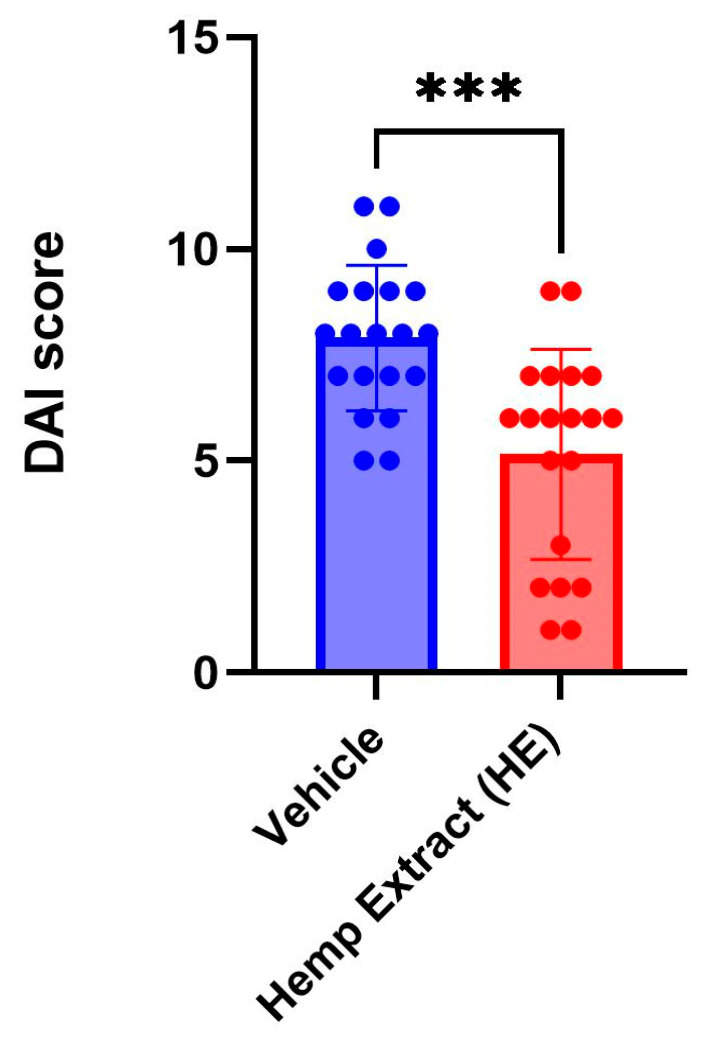
Disease activity index (DAI) scores measured on day 7 of the DSS treatment when inflammation is at the peak. The DAI scores in the cannabinoid-treated group are significantly lower than the vehicle-treated group (*n* = 20 mice/group, *** *p* < 0.005).

**Figure 3:**
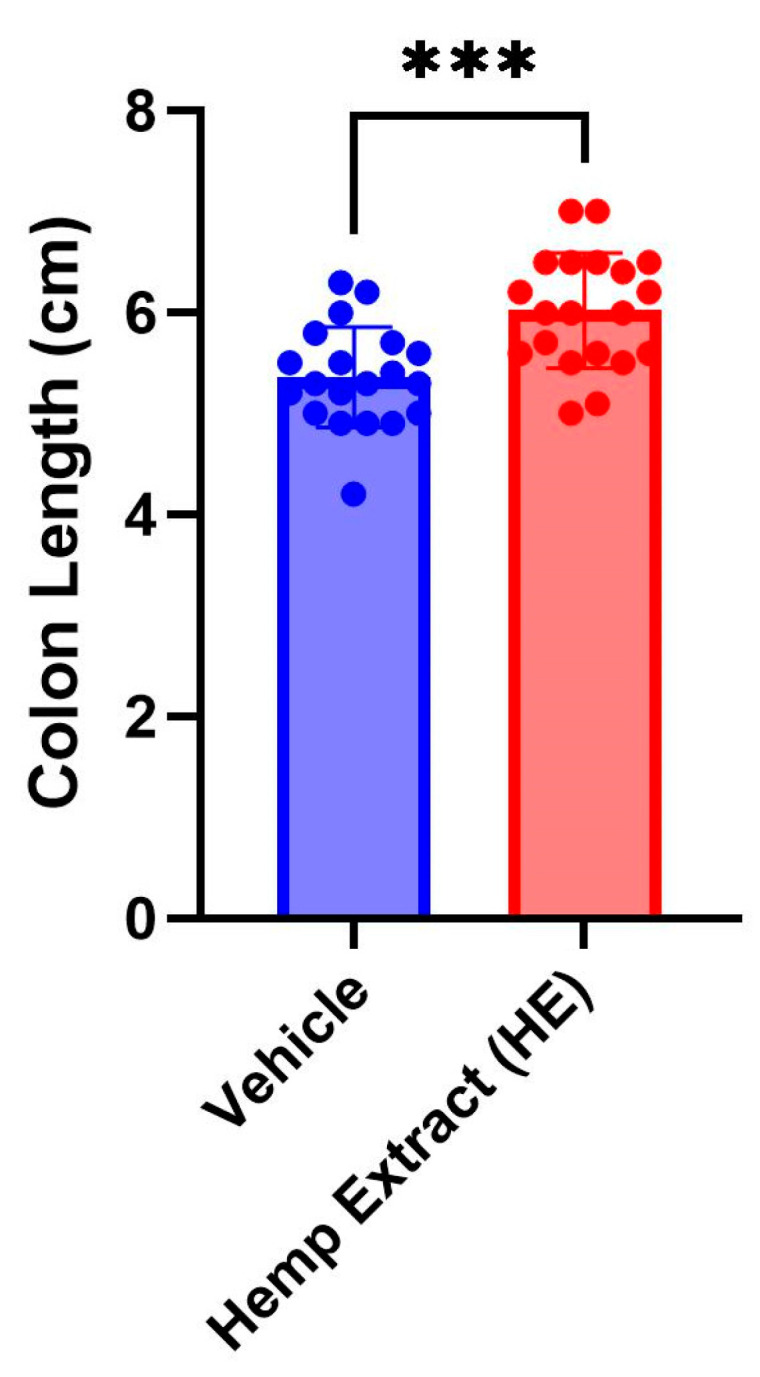
Colon length measured on day 7 of the DSS treatment when inflammation is at the peak. The colon lengths in the cannabinoid-treated group are significantly lower than the vehicle-treated group (*n* = 20 mice/group, *** *p* < 0.005).

The disease activity index (DAI) scores (% weight loss, stool consistency, and presence of fecal occult blood, Table 1) were assessed daily. The DAI scores were significantly lower in animals receiving HE compared to a vehicle (Figure 2). As a consequence of colitis, reduction of colon length is commonly observed in DSS-colitis mice. HE treatment helped in restoration of the colon length, aiding in recovery from UC (Figure 3). No changes were noted in colon length in animals that did not receive DSS but received either vehicle or hemp extract in Nutella (Supplementary Figure S1).

### 3.2. Oral Administration of CBD/CBG Hemp Extract Reduces Colonic Ulceration

**Figure 4:**
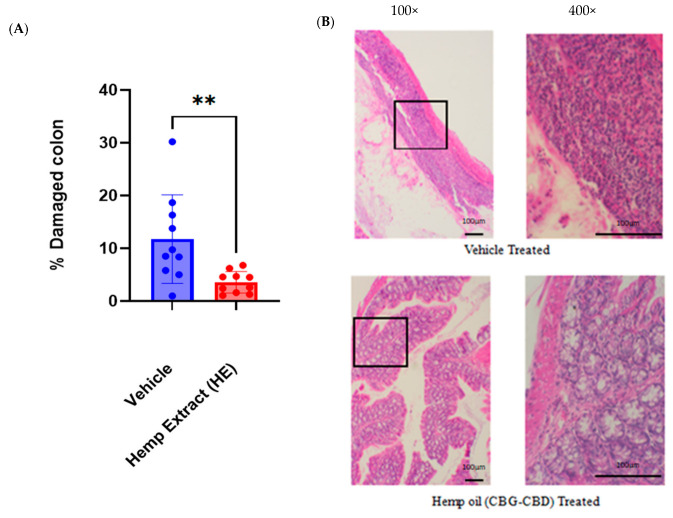
Colonic damage was assessed using H&E staining. (**A**) Colon damage (ulceration) was significantly lower for HE treated mice than control mice as measured by percentage of damaged epithelium. (**B**) Representative images (100×) of vehicle-treated and hemp oil-treated mouse colon highlighting damage to the colon; boxed region is shown at higher magnification (400×). (*n* = 10 mice/group, ** *p* < 0.01).

Histological scoring was performed on hematoxylin and eosin-stained Swiss roll sections to assess the damage incurred to the full-length colonic epithelium. Colons were collected on day 7 (2 days post DSS treatment) when the inflammation was at its peak. HE treatment limited the disruption of colonic morphology (loss of crypts) and reduced overall colonic damage significantly (Figure 4). Hemp extract did not impact colon morphology in non-colitic animals (Supplementary Figure S1).

### 3.3. Hemp Extract Treatment Helps in Restoration of Goblet Cells

**Figure 5:**
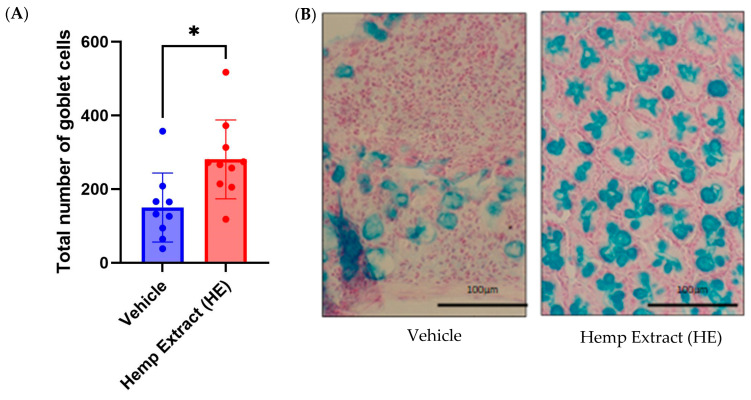
Hemp extract treatment increased goblet cell numbers. (**A**) The quantity of goblet cells is significantly higher in the HE-treated colitic mice compared to vehicle-treated. (**B**) Representative images (400×) of alcian blue stained colonic tissue from vehicle-treated and HE-treated mouse colon showing goblet cells (*n* = 10 mice/group, * *p* < 0.05).

The mucus layer on the intestinal epithelium is produced by goblet cells, and this mucus layer acts as a protection from pathogens. The depletion of goblet cells and reduction in the mucus layer are common to both DSS-induced colitis in mice and in UC patients. Murine colonic sections from vehicle or HE treated mice were stained with alcian blue, and the number of goblet cells was quantified. Relative to vehicle-treated animals, those treated with HE had a greater number of goblet cells (Figure 5).

### 3.4. Hemp Extract Administration Reduces Ulcerative Colitis Associated Proxy Measures of Abdominal Pain

**Figure 6:**
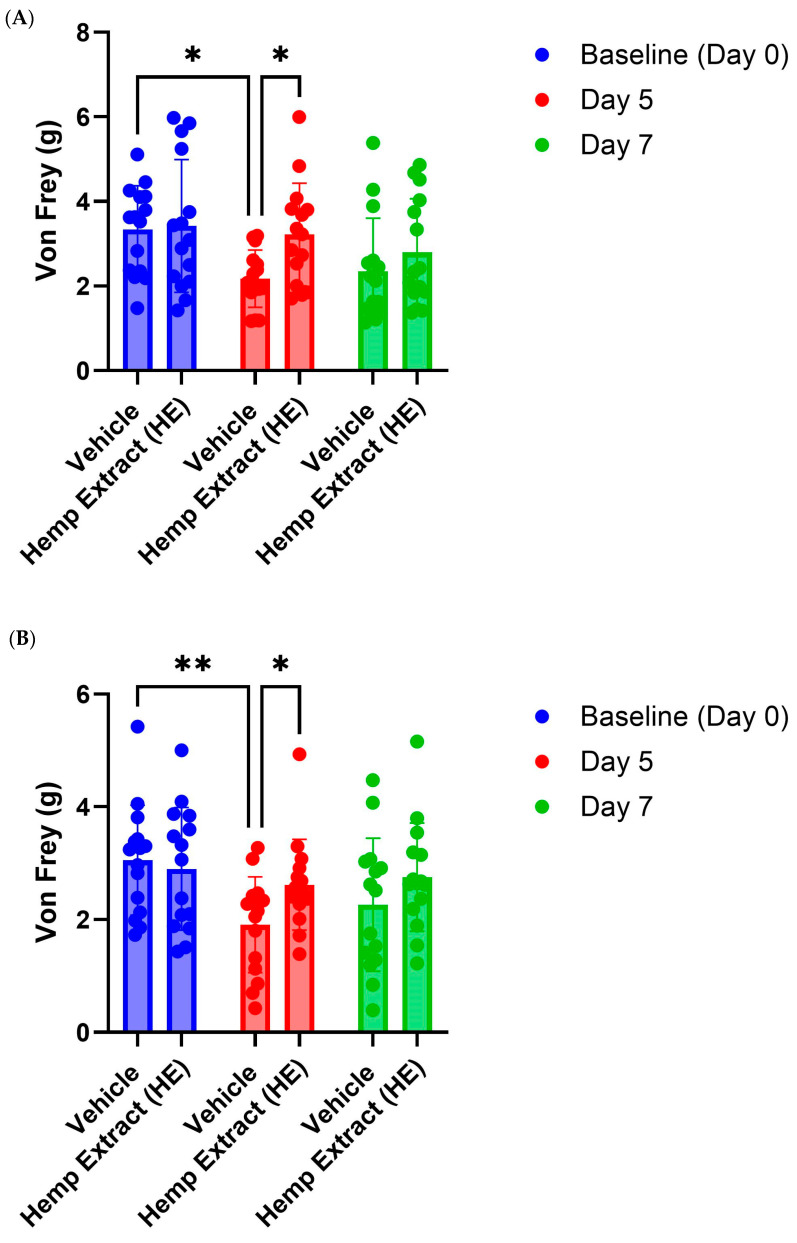
Abdominal wall hypersensitivity as measured using von Frey analysis. (**A**) Response in mice to abdominal von Frey assessment prior to DSS treatment, after 5 days of DSS treatment, and at tissue collection (2 days post DSS treatment) for vehicle and HE treated mice (administered orally). HE treatment reduced response to von Frey filament at the cessation of DSS but had no effect on day 7. (**B**) as in A except animals were treated via i.p. (*n* = 15 mice/group, * *p* < 0.05, ** *p* < 0.01).

Abdominal pain and visceral hypersensitivity are commonly observed in the DSS model of colitis as well as in UC patients [43]. Abdominal pain was assessed by measuring the response to mechanical stimuli, von Frey filament. Mice develop increased sensitivity to mechanical stimuli as the DSS treatment progresses and were the most sensitive on day 5 when the mice were taken off DSS treatment (Figure 5). HE treatment, administered via both diet and i.p., reduced abdominal pain in mice compared to mice that received vehicle (Figure 6). Mechanical hypersensitivity was also assessed in the right hind-paw; however, only HE administered orally was able to reduce somatic pain (Supplementary Figure S2).

### 3.5. Route of Administration Impact on Plasma Levels of CBG and CBD

**Figure 7:**
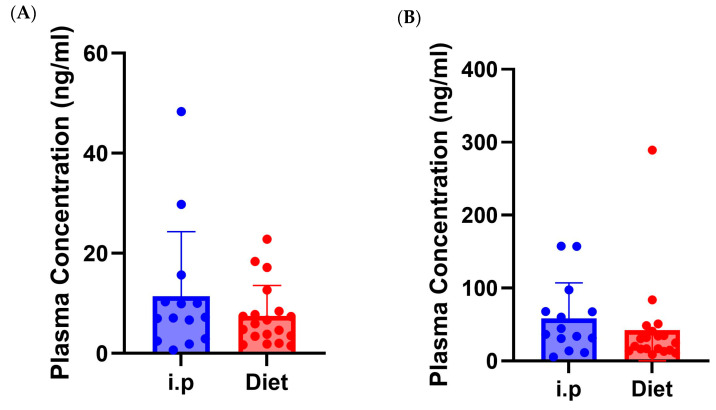
Plasma concentration of CBG and CBD. (**A**) Levels of cannabigerol (CBG) in plasma following 7 days of i.p. or oral delivery of hemp extract. (**B**) As in A, except cannabidiol levels are shown. (*n* = 20 mice/group).

Bioavailability of cannabinoids poses a challenge due to the hepatic metabolism of cannabinoids upon oral administration. To account for this, a slightly higher dose was administered for oral delivery experiments. Mass spectrometry analysis of plasma from HE animals showed no significant differences between i.p. or oral administration for either CBG or CBD (Figure 7). Interestingly, the levels of CBD were 5–10-fold higher than CBG regardless of route of administration; both compounds are in near equal levels in the HE used in this study.

## 4. Discussion

Using the DSS model of colitis, we demonstrate that a hemp extract high in CBG and CBD can help to improve inflammation and a proxy measure of abdominal pain. In particular, oral administration of HE results in reduced colonic damage, reduced disease activity index (DAI) scores, an increased number of goblet cells, and decreased abdominal wall sensitivity to mechanical stimulation. These findings are in line with our previous work showing that i.p. administration of HE reduces colonic damage [33]. The increased number of goblet cells observed in our current study may reflect the overall decrease in epithelial damage observed, or it may indicate that mice treated with HE have a faster recovery from DSS-induced epithelial damage. Interestingly, we noted a decrease in pain responses in mice treated with HE at the end of DSS treatment, but not on day 7 despite observing decreased tissue damage at this timepoint. Furthermore, only oral administration of HE was able to reduce somatic hyperalgesia in the hind paw. The mechanism by which HE reduces pain during and following colitis will need to be examined in future studies.

Plant-derived compounds are gaining popularity as therapeutic options due to the perception that these compounds are safer and have reduced side effects [44,45,46,47,48,49,50,51]. In particular, a large percentage of IBD patients report using cannabis or cannabis-based products to reduce and manage symptoms [22,51,52,53,54,55]. We demonstrate that using an HE high in CBD and CBG can prove to be an effective therapeutic option for management of UC symptoms as a result of their anti-inflammatory properties. CBG-CBD treatment not only provided overall symptomatic relief but also reduced colonic damage. A hallmark of colitis is the disruption of the epithelium and loss of crypt architecture along with loss of goblet cells leading to mucin layer defects [56,57,58]. We observe that cannabinoid treatment aids in restoration of epithelium by reducing immune cell infiltration. Although the impact of cannabinoids on immune cell migration is not yet elucidated, CBD is reported to reduce macrophage proliferation and reduce pro-inflammatory cytokine production [59,60,61]. CBG is also reported to reduce pro-inflammatory cytokine production and to reduce cellular damage by inhibiting reactive oxygen species (ROS) and inducible nitric oxide synthase (iNOS) production [62,63]. Consequently, numerous studies have corroborated the use of cannabinoids as anti-inflammatory agents and their use for treatment of immune-mediated disorders.

Previously, we reported improved disease activity scores and reduced epithelial damage following intraperitoneal administration of HE, and in the current study, we extend these findings to a more translatable route of delivery, self-administered oral delivery. Visceral pain and hyperalgesia are commonly associated with UC [64,65,66,67]. We found that HE reduced a proxy measure of abdominal pain following DSS administration, regardless of the route of HE administration, but did not impact pain levels in animals two days later. Cannabinoids, particularly CBD, CBG, and CBC (all components of the HE used), have been found to have analgesic activity in other models [25,68,69,70,71,72,73,74,75]. In other studies, CBG has been shown to reduce pain through interactions with CB1, CB2, and α2-adrenergic receptors and CBD through CB1, CB2 and serotonergic (5HT1a) receptors [76,77,78]. The mechanism by which CBC mediates analgesic activity remains unclear; however, it is known to be an agonist of TRPA1 and CB2, and activation of these receptors may be responsible [79,80,81,82].

It is important to note that cannabinoids undergo a high level of first pass metabolism and therefore have a lower bioavailability when administered orally [83,84,85]. To help account for this, a slightly higher dose of HE was administered orally (60 mg/kg total cannabinoid) compared to intraperitoneal injection (40 mg/kg total cannabinoid). The plasma concentration of CBG and CBD when administered via i.p and diet revealed no significant differences in the blood levels of cannabinoids, although slightly higher levels were detected following the lower dose i.p. than the higher oral dose. Interestingly, we observed that CBG levels were 5–10-fold lower compared to CBD levels, despite both being administered at nearly identical doses. This suggests that CBG is preferentially metabolized over CBD. CBD is known to be metabolized by CYP3A4, CYP2C9, and CYP2C19, while the metabolism of CBG is largely unknown. CBD has also been found to impact the metabolism of other medications [86,87,88,89]. Future studies will be needed to understand how CBD impacts the metabolism of CBG, but these findings may have broader implications for patients using hemp extracts, which are normally high in CBD, that are enriched for other phytocannabinoids.

While we did not directly assess the safety of administration of HE for purposes of treatment of UC during this study, existing safety data associated with the FDA-approved prescription CBD product Epidiolex^®^ suggest that HE use may prove to be a safer alternative for management of UC than the existing pharmaceutical treatments [90]. Oral administration of cannabinoids creates a translatable and patient-compliant method of cannabinoid administration for UC treatment. Further studies are necessary to refine our understanding of the impact of HE on inflammation, the mechanism by which HE reduces colitis, and the long-term effectiveness of HE in chronic colitis. Our current findings suggest that minor cannabinoids, particularly CBD and CBG, may prove to be useful, novel therapies for treating IBD. These findings are important because patients already report using cannabis and cannabinoid-based products for managing their symptoms, despite little clinical data to support the use of high-THC products for treating IBD [22,91,92]. In conclusion, our data suggest that HE enriched for CBD and CBG may offer therapeutic potential as a treatment for IBD patients.

## Figures and Tables

**Table 1:** Distribution of scores for measurement of disease activity index (DAI) scores for DSS model of colitis.

% Weight Loss		Stool Consistency		Hemoccult	
1–5%	1	Normal	0	Absent	0
6–10%	2	Soft/Loose	2	Hemoccult positive	2
11–20%	3	Diarrhea	4	Visible/Gross	4
>20%	4				

## Data Availability

All data are shown in manuscript, histology images from all tissue in the study are available via ScholarSphere.

## Supplementary Materials

The following supporting information can be downloaded at https://www.mdpi.com/article/10.3390/jcm14176095/s1: Figure S1: Colon lengths for control mice (receiving normal drinking water); Figure S2: Colon morphology (H&E staining) for control mice (receiving normal drinking water).

## Abbreviations

The following abbreviations are used in this manuscript:

IBDI	inflammatory bowel disease
UC	ulcerative colitis
CBD	cannabidiol
CBG	cannabigerol
HE	hemp extract
H&E	hematoxylin and eosin
