# Supplementary material for: Orally Administered CBD/CBG Hemp Extract Reduces Severity of Ulcerative Colitis and Pain in a Murine Model
Source: J Clin Med. 2025 Aug 28;14(17):6095. doi: 10.3390/jcm14176095 (PMC12429149; doi:10.3390/jcm14176095)
Supplement: Supplementary file 1 [file jcm-14-06095-s001.zip › jcm-3774276-supplementary.pdf]

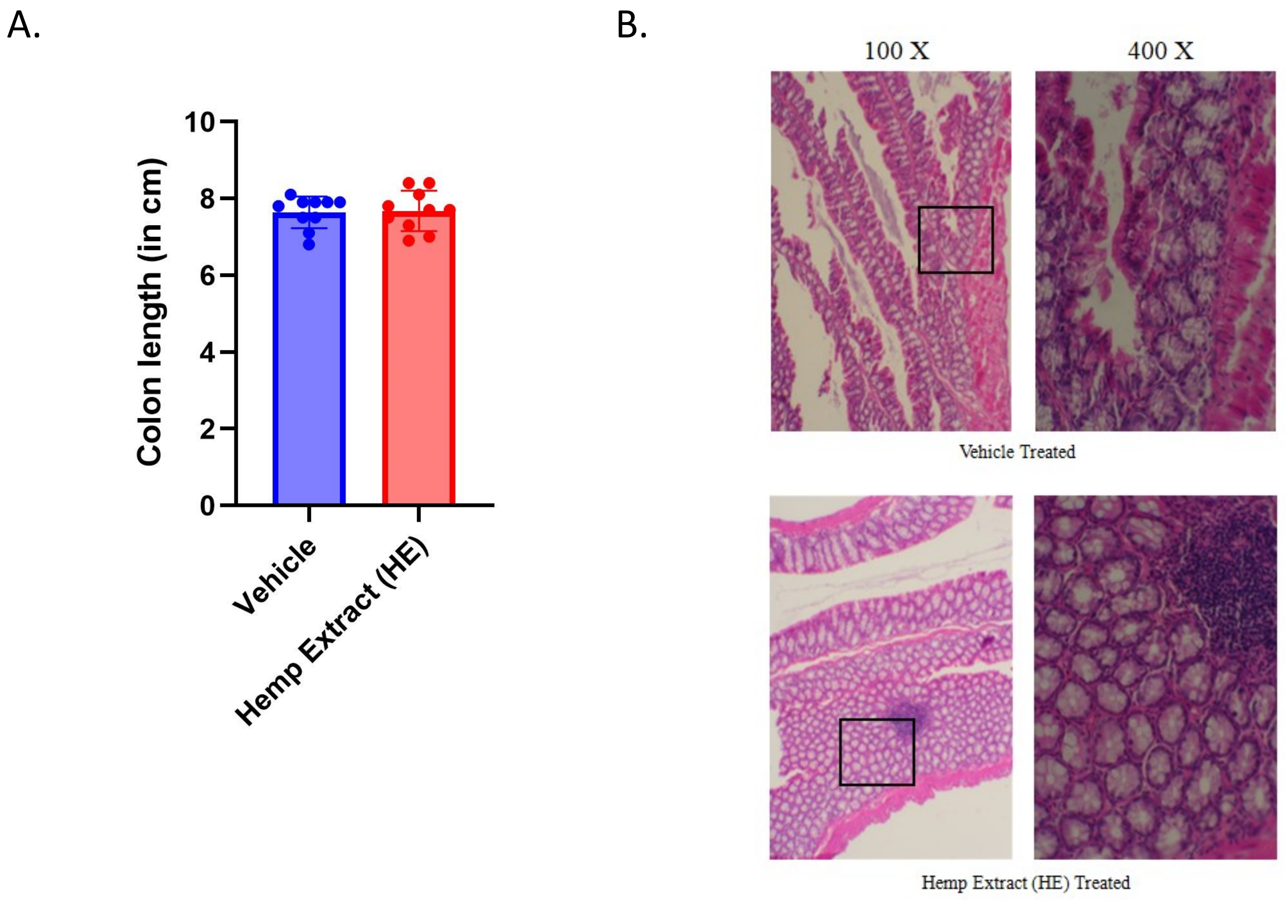

Supplemental Figure S1: Impact of Hemp Extract on Normal Colon. A) Colon length of mice fed vehicle (nutella) or HE in nutella. No differences in colon length were observed. B) Histological examination of colons from vehicle or HE fed animals. No differences in colonic architecture was observed with HE treatment.

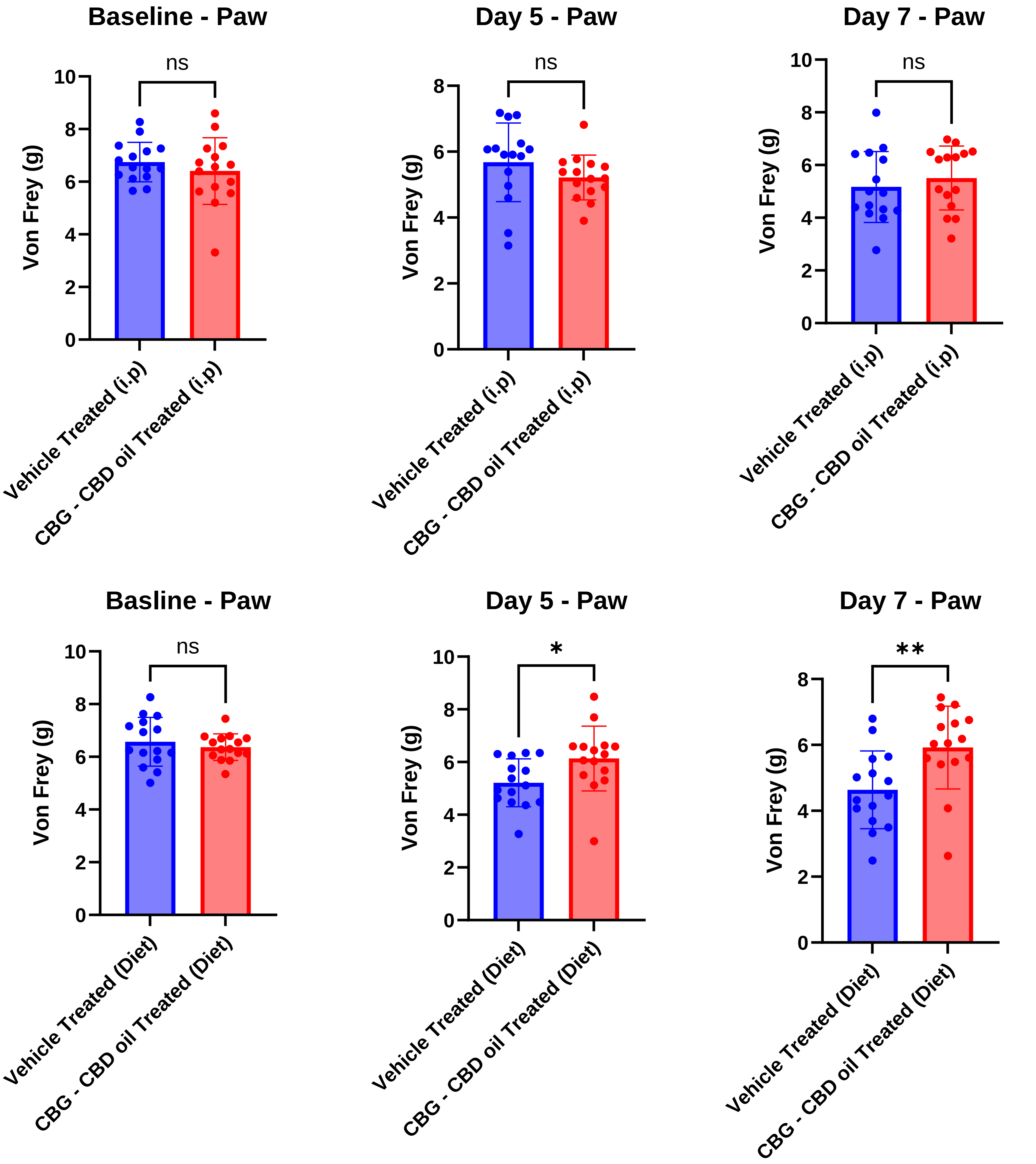

Supplemental Figure S2. Impact of Hemp Extract of Somatic Pain. von Frey testing of colitic animals of the hind paw in mice receiving hemp extract via i.p. or diet. Animals receiving HE in the diet following colitis, show reduced sensitivity to somatic pain compared to vehicle animals. This difference was not observed when HE was delivered via intraperitoneal injection.
